# The Role of Post-Translational Modifications in Regulation of NLRP3 Inflammasome Activation

**DOI:** 10.3390/ijms24076126

**Published:** 2023-03-24

**Authors:** Jing Xia, Songhong Jiang, Shiqi Dong, Yonghong Liao, Yang Zhou

**Affiliations:** 1 College of Veterinary Medicine, Southwest University, Chongqing 402460, China; 2 National Center of Technology Innovation for Pigs, Chongqing 402460, China

**Keywords:** inflammasome, NLRP3, post-translational modification

## Abstract

Pathogen-associated molecular patterns (PAMPs) and danger-associated molecular patterns (DAMPs) induce NLRP3 inflammasome activation, and subsequent formation of active caspase-1 as well as the maturation of interleukin-1β (IL-1β) and gasdermin D (GSDMD), mediating the occurrence of pyroptosis and inflammation. Aberrant NLRP3 inflammasome activation causes a variety of diseases. Therefore, the NLRP3 inflammasome pathway is a target for prevention and treatment of relative diseases. Recent studies have suggested that NLRP3 inflammasome activity is closely associated with its post-translational modifications (PTMs). This review focuses on PTMs of the components of the NLRP3 inflammasome and the resultant effects on regulation of its activity to provide references for the exploration of the mechanisms by which the NLRP3 inflammasome is activated and controlled.

## 1. Introduction

Pattern recognition receptors (PRRs) including NOD-like receptors (NLRs), Toll-like receptors (TLRs), RIG-I like receptors (RLRs) and C-type lectin receptors (CLRs) serve to detect pathogens and danger signals via sensing PAMPs and DAMPs to initiate innate immune responses, playing essential roles in host defense [1,2]. The NLR family pyrin domain containing 3 (NLRP3) is the sensor protein of the NLRP3 inflammasome which is extensively studied. However, its abnormal activation contributes to the development of a variety of diseases, such as Muckle–Wells syndrome, familial cold auto-inflammatory syndrome, systemic lupus erythematosus [3], neonatal-onset multisystem inflammatory disorder and rheumatoid arthritis [4]. Decreasing NLRP3 inflammasome activity is able to ameliorate many diseases, including gout [5], atherosclerosis [6], Alzheimer disease [7], traumatic brain injury [8] and stroke [9].

The NLRP3 inflammasome is a multiprotein complex, which consists of the sensor protein NLRP3, the adaptor apoptosis-associated speck-like protein containing a caspase activation and recruitment domain (ASC) and the effector caspase-1 [10]. NLRP3 is composed of an N-terminal pyrin domain (PYD), a central nucleotide-binding and oligomerization (NACHT) domain and a C-terminal leucine-rich repeat (LRR) domain. ASC, which contains an N-terminal PYD and a C-terminal caspase activation and recruitment domain (CARD), bridges NLRP3 with caspase-1 via homotypic PYD–PYD and CARD–CARD interactions [11]. Caspase-1 consists of three domains: an N-terminal CARD and catalytic subunits p10 and p20 [12].

PTM refers to irreversible or reversible covalent processing in some proteins after the translation [13]. It occurs at the amino acid side chains, C-terminus or N-terminus [14]. PTM changes the properties of amino acids by adding some particular chemical groups, proteins, carbohydrates or lipids to the amino acid side chains, or cleaving bonds enzymatically, which enhances the diversity of protein structures and functions [13]. These modifications are often induced by enzymatic catalysis, playing a critical regulatory role in physiological and pathological conditions [15]. Several PTMs are involved in the regulation of NLRP3 inflammasome activation, including ubiquitination, phosphorylation, SUMOylation, alkylation, S-nitrosylation, S-glutathionylation and acetylation [16]. This review focuses on the PTMs of the components of the NLRP3 inflammasome and the subsequent effects on its activity.

## 2. NLRP3 Inflammasome Activation

**Figure 1:**
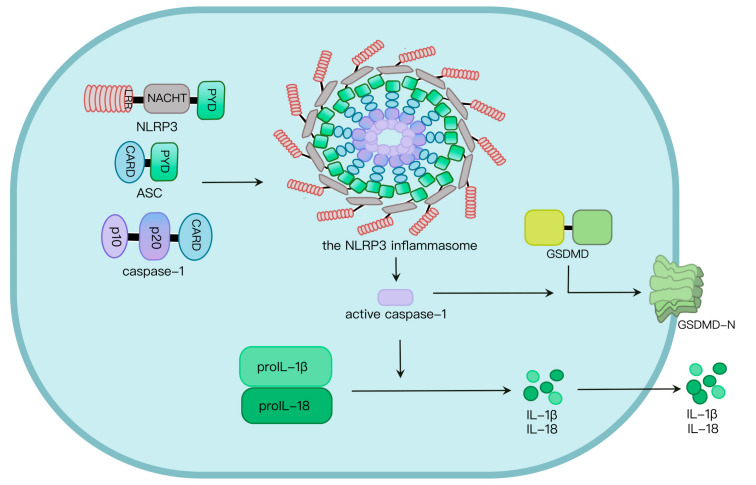
NLRP3 inflammasome activation. NLRP3, along with ASC, induces caspase-1 activation via assembly of the NLRP3 inflammasome upon challenges. Caspase-1 activation leads to maturation and secretion of proinflammatory cytokines such as IL-1β and IL-18, as well as programmed cell death called pyroptosis induced by GSDMD pores in the plasma membrane.

Two signals are required for NLRP3 inflammasome activation. Signal I involves the priming signal, induces IL-1β expression and upregulates NLRP3 expression through activating TLR and NF-κB pathways [17,18] as well as NLRP3 phosphorylation. In addition, signal II, transduced by PAMPs and host-derived DAMPs, triggers the assembly and activation of the NLRP3 inflammasome [19]. Mechanisms by which the NLRP3 inflammasome is activated are extensively explored. At least four models were proposed: K^+^ efflux [20], the generation of mitochondrial reactive oxygen species (mROS) [21], cathepsin B release from damaged lysosomes [22] and Ca^2+^ mobilization [23]. NLRP3 oligomerization via its NACHT domain leads to PYD clustering, which elicits recruitment and clustering of ASC through PYD–PYD interaction. ASC clustering subsequently provokes caspase-1 recruitment and assembly of the inflammasome complex. Then, caspase-1 undergoes autocleavage and formation of active p10/p20 tetramer, which cleaves proinflammatory cytokines such as IL-1β and IL-18 into their active molecules [24]. Active caspase-1 also cleaves GSDMD. The GSDMD N-terminal fragment (GSDMD-N) oligomerizes in the plasma membrane to generate approximately 21 nm-diameter GSDMD pores, leading to osmotic imbalance and cell death called pyroptosis (Figure 1) [24,25].

## 3. Regulation of Ubiquitination and Deubiquitination in NLRP3 Inflammasome Activation

Ubiquitin, a highly conserved small regulatory eukaryotic protein, contains 76 amino acids and 7 lysine residues, including K6, K11, K27, K29, K33, K48 and K63. It can be covalently attached to target proteins through a cascade of enzymatic reactions catalyzed by ubiquitin-activating enzymes (E1), ubiquitin-conjugating enzymes (E2) and ubiquitin ligases (E3) [26]. Ubiquitin is bound to the target substrates via an isopeptide bond formed between the C-terminal glycine of ubiquitin and the ε-amino group of lysine in the substrate [27]. Similar isopeptide bonds can be formed from linkage of the C-terminus of one ubiquitin to one of the seven lysine residues or the N terminal methionine on another ubiquitin to form ubiquitin chains [28]. Deubiquitinases remove conjugated ubiquitin from the substrates [29]. Many proteins are involved in the ubiquitination and deubiquitination of NLRP3 inflammasome components to regulate inflammasome activity (Table 1). Recent advances in the development of pharmacological targeting of ubiquitination and deubiquitination have uncovered a great potential for treatments of cancer, neurodegenerative disorders, inflammatory disorders, immunological diseases and microbial infection [30]. YTH N6-methyladenosine (m6A) RNA-binding protein 1 (YTHDF1), a reader of m6A, alleviates cecal ligation and perforation-induced sepsis through promoting NLRP3 ubiquitination [31]. Tranilast blunts NLRP3 inflammasome assembly via enhancing NLRP3 ubiquitination, contributing to the amelioration of vascular inflammation and atherosclerosis [32].

### 3.1. Ubiquitination of NLRP3

NLRP3 ubiquitination plays a critical role in the regulation of its activity. The effects of different types of ubiquitin chains on the NLRP3 activity vary. Among the three enzymes participating in ubiquitination, E3 ubiquitin ligases act as the major proteins involved in the orchestration of NLRP3 inflammasome activity. Its regulation in NLRP3 inflammasome activation is mainly mediated by K48-, K63- and K27-linked ubiquitination. K48-linked ubiquitin chains employ a closed conformation with the hydrophobic residues at the interdomain interface exposed to ubiquitin chain recognition factors, serving as a signal for degradation by the 26S proteasome [51]. K63-linked ubiquitin chains act as a signal for altering the functions of the modified protein, including signaling transduction, DNA repair and intracellular trafficking [52].

K48-linked ubiquitination inhibits NLRP3 inflammasome activation. This may be caused by degradation of NLRP3. It seems that K48-linked ubiquitination plays a more predominant role than K63-linked ubiquitination. Ubiquitin-specific peptidase 5 (USP5), a scaffold protein, recruits membrane-associated ring-CH-type finger 7 (MARCH7) to NLRP3, and mediates K48-linked ubiquitination of the LRR domain of NLRP3 and its autophagic degradation [33,53]. Ariadne homolog 2 (ARIH2) engages in the regulation of protein degradation [54,55,56]. Interaction between the ARIH2 and NACHT domain of NLRP3 induces K48- and K63-linked ubiquitination, restraining NLRP3 inflammasome activation [36]. The E3 ubiquitin ligase ring-finger protein 125 (RNF125) recruits Casitas-B-lineage lymphoma protein-b (Cbl-b) after initiating K63-linked ubiquitination of the NLRP3 LRR domain. Cbl-b binds to the K63-linked ubiquitin chains via its ubiquitin-associated region (UBA), and induces K48-linked ubiquitination of NLRP3 at the Lys496 site and subsequent proteasome-mediated degradation [34,57]. Tripartite motif (TRIM) proteins consist of a large family of E3 ubiquitin ligases containing a RING finger domain, one or two B-box domains and a coiled-coil domain [58]. TRIM31 interacts with NLRP3 PYD, mediating K48-linked ubiquitination of NLRP3 and its degradation [35]. Inconsistent with this finding, Tang and colleagues suggested that TRIM31 does not interact with NLRP3 PYD [34]. TRIM65 binds to the NLRP3 NACHT domain, causes K48- and K63-linked ubiquitination of NLRP3 and inhibits the interaction between the mitotic kinase NIMA-related kinase 7 (NEK7) and NLRP3, thereby negatively regulates NLRP3 inflammasome activation [37]. NEK7 is a critical mediator of NLRP3 inflammasome activation. It facilitates the oligomerization and activation of NLRP3 [59,60].

In contrast to K48-linked ubiquitination of NLRP3, K63-linked ubiquitination licenses the inflammasome activation on most occasions. The E3 ubiquitin ligase pellino-2 [38] and E2 ubiquitin-conjugating enzyme 13 (Ubc13) [40] induce the K63-linked ubiquitination of NLRP3, which positively regulates NLRP3 inflammasome activity. Pellino-2 mediates NLRP3 ubiquitination and its activation following treatment with lipopolysaccharide (LPS) [38]. Ubc13 functions as a K63-specific ubiquitin-conjugating enzyme [61]. It interacts with and ubiquitinates NLRP3 at Lys565 and Lys687, contributing to NLRP3 inflammasome activation [40]. Inconsistent with these findings, cullin1, an essential component of the Skp1-Cullin1-F-box E3 ligase, inhibits NLRP3 inflammasome activation via induction of K63-linked ubiquitination of NLRP3 at Lys689 [39]. The mechanisms by which K63-linked ubiquitination influences NLRP3 inflammasome activation is unclear. NLRP3 undergoes intracellular trafficking during NLRP3 inflammasome assembly. Under the resting conditions, exogenous and most endogenous NLRP3 resides on the endoplasmic reticulum in THP-1 macrophages and murine bone marrow-derived macrophages (BMDMs), respectively [62,63], and exogenous NLRP3 is predominantly cytosolic in HEK293T cells and BMDMs [64]. Upon stimulation with NLRP3 agonists, the trans-Golgi network (TGN) disassembles from perinuclear clusters into vesicles. NLRP3 redistributes to mitochondria-associated endoplasmic reticulum membranes (MAMs) at the perinuclear region [63]. MAMs cluster close to TGN in an NLRP3-independent mechanism, supporting NLRP3 aggregation on dispersed TGN via ionic bonding between its conserved polybasic region (aa127–146) in the PYD-NACHT polybasic linker and negatively charged phosphatidylinositol-4-phosphate (PtdIns4P) on the dispersed TGN, where NLRP3 is phosphorylated at Ser293 by protein kinase D (PKD) [65,66]. Subsequently, phosphorylated NLRP3 undergoes oligomerization and deubiquitination, enters the cytosol and forms the inflammasome with ASC and caspase-1 [66]. Whether K63-linked ubiquitination is associated with intracellular trafficking of NLRP3 still needs to be explored.

K27-linked ubiquitination of NLRP3 induced by various E3 ubiquitin ligases differentially regulates NLRP3 inflammasome activation. K27-linked ubiquitination of NLRP3 NACHT domain at Lys380 induced by the β-transducin repeat-containing E3 ubiquitin protein ligase 1 (β-TrCP1) increases the proteasomal degradation and prevents NLRP3 inflammasome activation [41]. Conversely, HECT, UBA and WWE domain-containing E3 ubiquitin protein ligase 1 (HUWE1) interacts with NLRP3 to mediate its K27-linked ubiquitination, facilitating NLRP3 inflammasome activation [42].

Some E3 ubiquitin ligases regulate NLRP3 inflammasome activation via an unknown linkage type of ubiquitin chains. Parkin RBR E3 ubiquitin protein ligase (PARK2) inhibits NLRP3 inflammasome activation through interacting with and ubiquitinating NLRP3 [67]. F-box and leucine-rich repeat protein 2 (FBXL2), a member of the Skp-Cullin-F box (SCF) family, is involved in NLRP3 ubiquitination at Lys689 and its degradation via interaction with NLRP3 at Trp73 [43].

### 3.2. Deubiquitination of NLRP3

Deubiquitinases remove K48- and K11-linked ubiquitin chains, contributing to NLRP3 inflammasome activation. This is consistent with the effect of K48-linked ubiquitination on NLRP3 inflammasome activity. Upon infection with the DNA virus herpes simplex virus type 1 (HSV-1), the stimulator of interferon genes (STING) binds to NLRP3, attenuates K48- and K63-linked ubiquitination of NLRP3 and increases its protein expression on the endoplasmic reticulum, facilitating NLRP3 inflammasome activation and the subsequent release of proinflammatory cytokines [44]. Ubiquitin-specific peptidase 1 (USP1)-associated factor 1 (UAF1, also called WDR48 or p80) is a stoichiometric binding partner of USP1 [68]. The UAF1-USP1 deubiquitinase complex selectively eliminates K48-linked ubiquitin chains of NLRP3 to stabilize its expression via interaction with the LRR and NACHT domains, which in turn promotes NLRP3 inflammasome activation [45]. 

Although K63-linked ubiquitination of NLRP3 supports the inflammasome activation in most settings, removal of K63-linked ubiquitin chains induced by different deubiquitinases plays distinct regulatory roles in NLRP3 inflammasome activation. Ubiquitin C-terminal hydrolase L5 (UCHL5) contributes to the inflammasome activation via elimination of K63-linked ubiquitin chains on NLRP3 [46]. Abraxas brother 1 (ABRO1), a subunit of the BRCA1/BRCA2-containing complex subunit 36 (BRCC36) isopeptidase complex (BRISC), impedes WW domain-containing E3 ubiquitin-protein ligase 2 (WWP2)-mediated BRCC3 ubiquitination and increases BRCC3 stability [69]. BRCC3 deubiquitinates NLRP3 LRR domain and positively regulates NLRP3 inflammasome activation [70]. Following stimulation with LPS, ABRO1–NLRP3 interaction promotes the inflammasome activation via removal of K63-linked ubiquitin chains on NLRP3 [71]. Inconsistent with these deubiquitinases, the signal transducing adaptor molecule-binding protein (STAMBP) eliminates K63-linked ubiquitin chains and inhibits inflammasome activation [47]. 

### 3.3. Ubiquitination and Deubiquitination of ASC and Caspase-1

The influence of K63-linked chains on ASC still remains to be investigated. Both the addition and elimination of K63 ubiquitin chains promote NLRP3 inflammasome activation. This may be associated with different modification sites or regulation by other signaling pathways triggered by ubiquitin enzymes or deubiquitinases. On the one hand, mitochondrial antiviral signaling protein (MAVS) facilitates interaction between the tumor necrosis factor receptor-associated factor (TRAF3) and ASC, provoking K63-linked ubiquitination of ASC at Lys174 and contributing to NLRP3 inflammasome activation [48,72]. Pellino E3 ubiquitin protein ligase 1 (Peli1) mediates both K48- and K63-ubiquitination [73,74]. It enhances ASC oligomerization and NLRP3-ASC interaction by conjugating K63 ubiquitin chains to ASC at Lys55, facilitating the NLRP3 inflammasome activation [49]. On the other hand, USP50 removes K63-linked ubiquitin chains of ASC, which promotes the NLRP3 inflammasome activation [75]. USP7 which cleaves K48- and K63-linked chains is involved in ASC oligomerization and speck formation, promoting NLRP3 inflammasome assembly [76,77]. 

In addition, TRAF6 [78], USP3 [79] and USP8 [50] also play regulatory roles in NLRP3 inflammasome activation. Far-infrared suppresses NLRP3 inflammasome activation through increasing TRAF6 expression and inducing ASC ubiquitination [78]. USP3 removes K48-linked ubiquitin chains of ASC and improves its stability by blocking proteasomal degradation to promote NLRP3 inflammasome activation [79]. USP8 eliminates K11-linked ubiquitin chains of caspase-1 at Lys134 to prevent its degradation, contributing to NLRP3 inflammasome activation [50].

## 4. Regulation of Phosphorylation and Dephosphorylation in NLRP3 Inflammasome Activation

Phosphorylation modulates protein function and controls the turnover of its targets and subcellular localization by altering protein conformation or influencing protein–protein interaction. Protein kinases mediate the phosphate group transfer from ATP to serine, threonine and tyrosine residues of the substrates, while phosphatases removes the phosphate group of a phosphorylated protein substrate [80]. Phosphorylation and dephosphorylation of the components of the NLRP3 inflammasome control its activity. Several proteins are involved in phosphorylation and dephosphorylation of NLRP3 inflammasome components to regulate inflammasome activity (Table 2).

### 4.1. NLRP3 Phosphorylation

NLRP3 phosphorylation at Ser725 [81], Ser194 [82] or tyrosines in the PYD-NACHT polybasic linker [89] contribute to NLRP3 inflammasome activation. Misshapen-like kinase 1 (MINK1), a member of the mammalian germinal center kinase (GCK) family [90], binds to the NLRP3 LRR domain, and phosphorylates NLRP3 at Ser725, which are critical for the priming step of NLRP3 inflammasome activation [81]. ROS serves only as a priming signal, but fails to contribute to the activation step of the NLRP3 inflammasome [91]. It is able to increase the kinase activity of MINK1 and facilitate NLRP3 phosphorylation at Ser725. This subsequently promotes inflammasome priming [81]. The C-Jun N-terminal kinase 1 (JNK1) directly phosphorylates NLRP3 at Ser194 to facilitate NLRP3 deubiquitination and oligomerization, which is an essential priming event for inflammasome activation. Cryopyrin-associated periodic syndromes (CAPS) are caused by gain-of-function mutations of NLRP3, and blocking NLRP3 phosphorylation by S194A mutation abolishes LPS-induced CAPS-associated inflammasome activation [82]. Bruton tyrosine kinase (BTK) directly binds to NLRP3 and phosphorylates four tyrosine residues in the PYD-NACHT polybasic linker, including Tyr132, Tyr136, Tyr145 and Tyr164. It causes charge neutralization of the polybasic region peptide sequence, relocalization from intact TGN to dispersed TGN and oligomerization of NLRP3, ASC polymerization and NLRP3 inflammasome assembly [89]. Of note, the effect of BTK activity on NLRP3 inflammasome activation is related to the dose of LPS. BTK prevents NLRP3 inflammasome activation upon priming with a high dose of LPS, and plays a positive regulatory role upon priming with a low dose. This is due to the impaired TLR4-mediated responses and insufficient activation following stimulation with a low dose in BTK-KO cells [92]. 

NLRP3 phosphorylation at Ser5 restricts NLRP3 inflammasome activation. The serine/threonine kinase AKT, also called protein kinase B (PKB), interacts with NLRP3 LRR domain via its central kinase domain (aa150–408), leading to its phosphorylation at Ser5, as well as inhibition of NLRP3 oligomerization and ASC recruitment [93]. 

Different types of kinase-mediated phosphorylation at Ser295 in human NLRP3 (mouse Ser293) play distinct roles in the regulation of the inflammasome activity. Both PKD [66] and protein kinase A (PKA) [86] phosphorylate human NLRP3 at Ser295. In response to NLRP3 agonists, NLRP3 enters the cytosol after phosphorylation by PKD, allowing for inflammasome assembly [66]. PKD inhibition with the specific inhibitor CRT 0066101 led to reduced NLRP3 inflammasome activity in LPS-stimulated peripheral blood mononuclear cells (PBMCs) isolated from CAPS patients [66]. ATP hydrolysis is required for NLRP3 self-association and inflammasome assembly. PKA is rapidly activated by elevated intracellular levels of cyclic adenylyl monophosphate (cAMP), and phosphorylates NLRP3 at Ser295 to turn off its ATPase [86]. Meantime, PKA also mediates K48- and K63-linked ubiquitination of NLRP3, eliciting the suppression of inflammasome activity [94].

### 4.2. NLRP3 Dephosphorylation

NLPR3 dephosphorylation at Ser5 [84], Ser803 [95] or Tyr861 [85] enhances NLRP3 inflammasome activation. The phosphatase protein, phosphatase 2A (PP2A), dephosphorylates NLRP3 at Ser5 to allow its activation, and the regulation of PP2A in NLRP3 inflammasome activation is controlled by BTK [84,96]. Protein tyrosine phosphatase non-receptor type 22 (PTPN22) interacts with and dephosphorylates NLRP3 at Tyr861, decreasing NLRP3 inflammasome-mediated IL-1β secretion [85]. In the resting state, NLRP3 is predominantly membrane bound, which facilitates 12–16 molecules to form double-ring cage structures by LRR–LRR interactions with PYDs shielded within NACHT-LRR rings. NACHT domains in the cage are hardly in contact. NEK7 disrupts the NLRP3 cage to enable its conformational rearrangement. Dispersion of intact TGN into vesicles, an early event for NLRP3 activators, and inflammasome assembly are dependent on the double-ring cage structure. The priming signal induces mouse NLRP3 Ser803 (Ser806 in human NLRP3) phosphorylation at the LRR domain, and signal II triggers dephosphorylation at Ser803 to enable NEK7 binding. Ser803 localizes adjacent to positively charged residues of the neighboring LRR. Ser803 phosphorylation may support TGN dispersion by stabilizing the double-ring cage structure [97]. Additionally, phosphomimetic substitutions of Ser803 impair the NEK7 recruitment to NLRP3 in vitro and in vivo, as well as the BRCC3-mediated NLRP3 deubiquitination [95,98]. Casein kinase 1 alpha 1 (CSNK1A1) serves as the key kinase that targets NLRP3 phosphorylation at Ser803 [95]. 

### 4.3. Phosphorylation of ASC and Caspase-1

The phosphorylation and dephosphorylation of NLRP3 at different sites play distinct roles in the regulation of NLRP3 inflammasome activation, while the phosphorylation of ASC and caspase-1 promotes NLRP3 inflammasome activation. Pyruvate kinase (Pyk2) [87] and JNK [99] are involved in human ASC Tyr146 (mouse ASC Tyr144) phosphorylation, facilitating ASC oligomerization and NLRP3 inflammasome assembly. Spleen tyrosine kinase (Syk) contributes to NLRP3 agonist-mediated ASC self-association and inflammasome assembly through phosphorylating ASC at Tyr146 and Tyr187 residues [99,100]. LPS treatment induces interaction between p21 (Rac family small GTPase 1) activated kinase 1 (PAK1) and caspase-1, mediating caspase-1 phosphorylation at Ser376 and its activation [88].

## 5. Regulation of SUMOylation in NLRP3 Inflammasome Activation

The small ubiquitin-like modifier (SUMO) protein is evolutionarily conserved and ubiquitously expressed in eukaryotes. It belongs to the ubiquitin-like family and alters the properties and functions of modified proteins via PTMs [101,102,103]. Four SUMO proteins have been identified in humans, SUMO1–4. SUMO2 and -3 are highly homologous [104]. SUMOylation is a reversible PTM process. SUMO binds to a lysine residue of a substrate and is removed from the modified protein through SUMO-specific peptidase-mediated deSUMOylation. SUMO is expressed as a C-terminally extended precursor, and then processed to generate the active form. The SUMO-activating enzyme SAE1/SAE2 covalently links to the C-terminus of SUMO via the sulfhydryl group of a cysteine residue; then, SUMO is transferred to the SUMO-conjugating enzyme Ubc9, and finally conjugated to a lysine side chain of the target protein mediated by a SUMO ligase. SUMO chains are able to assemble on substrates [105,106].

NLRP3 Lys204 SUMOylation facilitates its activation [107], while Lys689 SUMOylation suppresses its activation [108]. Ubc9 binds to NLRP3 to mediate its SUMOylation at Lys204 induced by SUMO1, which facilitates ASC oligomerization and inflammasome assembly. Interaction between SUMO-specific protease 3 (SENP3) and NLRP3 results in NLRP3 deSUMOylation and attenuates ASC speck formation, as well as inflammasome activation [107]. Mitochondrial-anchored protein ligase (MAPL), also called mitochondrial E3 ubiquitin protein ligase 1 (MUL1), mediates RING-finger-dependent NLRP3 Lys689 SUMOylation, and enhances caspase-1 maturation and IL-1β release in murine macrophages and human cells. NLRP3 Lys689 deSUMOylation induced by SENP6 and 7 promotes NLRP3-dependent ASC oligomerization and caspase-1 activation. MAPL has no effect on absent in melanoma 2 (AIM2) inflammasome activation [108]. Additionally, SUMOylation can also orchestrate protein function via influencing ubiquitination [109]. TRIM28 favors NLRP3 SUMOylation to inhibit its ubiquitination and proteasome-mediated degradation, contributing to NLRP3 inflammasome activation [16]. 

## 6. Regulation of Alkylation in NLRP3 Inflammasome Activation

Alkylation targeting ATPase activity of NLRP3 negatively controls the inflammasome activation through a decreasing affinity for ATP. The NACHT domain, carrying ATPase activity, adopts an ADP/ATP switch mechanism to orchestrate NLRP3 activation [110]. The NACHT domain is composed of four subdomains, including a nucleotide-binding domain (NBD, aa131–373 in human NLRP3), a helical domain 1 (HD1, aa374–434), a winged-helix domain (WHD, aa435–538) and helical domain 2 (HD2, aa539–676). Interaction between WHD His522 and ADP keeps NLRP3 in a closed and inactive conformation [111,112]. Treatment with NLRP3 agonists leads to the exit of ADP, the binding of ATP with the Walker A motif in NBD and ATP hydrolysis [110]. MCC950 suppresses ATP hydrolysis and NLRP3 inflammasome activation via interaction with the Walker B motif in NBD [113]. 3, 4-methylenedioxy-β-nitrostyrene (MNS) [114], 2-cyclohexylimino-6-methyl-6,7-dihydro-5H-benzo [1,3], oxathiol-4-one (BOT-4-one) [115] and vanillylacetone [116] mediate NLRP3 alkylation and reduce its ATPase activity. The nitrovinyl group of MNS plays an essential role in its inhibitory activity. MNS restricts NLRP3-mediated ASC oligomerization in potassium efflux- and Syk-independent manners, and does not influence the activation of the NLR family CARD domain-containing 4 (NLRC4) or AIM2 inflammasomes [114]. BOT-4-one suppresses L540 lymphoma cell survival and proliferation, and may have therapeutic potential in human cancer therapy, especially Hodgkin’s lymphoma [117]. Application of BOT-4-one alleviates 2,4,6-trinitrochlorobenzene-induced contact and atopic dermatitis, IL-23-induced psoriasis-like skin inflammation [118] and collagen-induced arthritis in mice [119]. Additionally, BOT-4-one enhances NLRP3 ubiquitination, which may exert inhibitory effects on the inflammasome activation [115]. The IκB kinase-β inhibitor Bay 11–7082 and structurally related vinyl sulfone compounds inactivate caspase-1 by direct alkylation of several conserved cysteines on the p20 subunits, including Cys285, Cys136, Cys169 and Cys244. They also show an inhibitory effect on the NLRC4 inflammasome [120].

NLRP3 itaconation inhibits NLRP3 inflammasome activation. Itaconate, an unsaturated C5-dicarboxylic acid, is produced by the decarboxylation of the Krebs cycle intermediate cis-aconitate catalyzed by cis-aconitate decarboxylase (ACOD1) that is encoded by immuno-responsive gene 1 (Irg1) [121]. Both itaconate and its derivative 4-octyl itaconate (4-OI) have been shown to directly alkylate cysteine residues on target proteins. Treatment of LPS-primed BMDMs with 4-OI prior to ATP or nigericin dicarboxypropylates Cys548 in NLRP3 HD2 interferes with the NLRP3–NEK7 interaction, and inhibits caspase-1 maturation and IL-1β secretion. Nigericin triggers enhanced caspase-1 maturation and IL-1β secretion in LPS-primed *Irg1*^-/-^ BMDMs. 4-OI specifically regulates NLRP3 inflammasome activity, and has no effect on the AIM2, NLRC4 or non-canonical inflammasome [121,122]. ASC oligomerization is enhanced in Irg1^-/-^ BMDMs that is retreated with LPS + ATP or LPS + nigericin after 24 h of LPS pretreatment [123]. Whether itaconate regulates NLRP3 inflammasome activation via succinate dehydrogenase (SDH) is debatable. Lampropoulou and colleagues found that dimethyl malonate, a SDH inhibitor, inhibited LPS (100 ng/mL, 4 h) +ATP (3 mM, 45 min)-induced IL-1β release in BMDMs [124], while Hooftman and colleagues’ study showed that dimethyl malonate fails to influence IL-1β secretion in LPS (100 ng/mL, 3 h) +ATP (5 mM, 45 min)-treated BMDMs [121].

## 7. Regulation of S-Nitrosylation in NLRP3 Inflammasome Activation

Similar to NLRP3 alkylation, NLRP3 S-nitrosylation plays an inhibitory role in the inflammasome activity. S-nitrosylation refers to the covalent binding of a NO group to a protein cysteine thiol to form S-nitrosothiols [125]. S-nitroso-N-acetylpenicillamine (SNAP), an NO donor, dampens nigericin-induced capase-1 maturation, as well as release of IL-1β and IL-18, in the TLR1/2 agonist PAM3CSK4-primed murine peritoneal macrophages. Priming with PAM3CSK4 causes S-nitrosylation of NLRP3 and caspase-1, and the C-terminus of NLRP3 is more susceptible to S-nitrosylation than its N-terminus. Inflammasomes of AIM2 and NLRC4 are partially inhibited [126]. HEK293T cells transfected with NLRP3 or caspase-1 expression plasmids were treated with SNAP or not, and lysed to obtain lysate 1. Independent cultures transfected with plasmids expressing the other components of the NLRP3 inflammasome and IL-1β were lysed to obtain lysate 2. Mature IL-1β was assessed in the mixed lysates with lysate 1 and lysate 2. The results displayed that IL-1β processing was inhibited only in the SNAP treatment of NLRP3-expressing cells, but not in the SNAP treatment of caspase-1-expressing cells, indicating that S-nitrosylation of NLRP3, rather than that of caspase-1, is sufficient to regulate NLRP3 inflammasome activity [127]. The mechanisms by which NLRP3 S-nitrosylation affects the inflammasome activity still needs to be clarified. Repressing NLRP3 inflammasome activity via the S-nitrosylation of caspase-1 suppresses angiogenesis, invasion and metastasis of melanoma and breast cancer cells [128].

## 8. Regulation of Acetylation in NLRP3 Inflammasome Activation

In contrast to alkylation and S-nitrosylation of NLRP3, NLRP3 acetylation boosts the inflammasome activation. Lysine acetyltransferase 5 (KAT5), also called Tat-interactive protein 60 kDa (TIP60), belongs to MOZ-Ybf2/Sas3-Sas2-TIP60 (MYST) family [129]. KAT5 mediates NLRP3 acetylation at Lys24, facilitating its interaction with NEK7 and oligomerization. The KAT5 inhibitor NU9056 restricts NLRP3-dependent caspase-1 maturation and IL-1β release both in vivo and in vitro [130]. Sirtuin 2 (SIRT2), an NAD^+^-dependent deacetylase and a metabolic sensor, targets NLRP3 for deacetylation in BMDMs. *Sirt2* deletion or treatment with the SIRT2 inhibitor AGK2 results in increased IL-1β production and cleaved caspase-1, but has no effect on pro-IL-1β expression. *Sirt2* knockout makes no change to caspase-1 maturation and IL-1β secretion following stimulation with flagellin, a NLRC4 inducer, or poly(dA:dT), an AIM2 inducer. Compared to WT mice, *Sirt2*^-/-^ mice fed a high-fat diet for 6 months or fed a chow diet for 2 years promoted NLRP3 inflammasome activation, accumulated more body fat and displayed increased levels of plasma glucose and insulin. K21/22/24 at the acetylation sites of NLRP3 were mutated to arginine to mimic the constitutively deacetylated state. Aged *Nlrp3*^-/-^ mice reconstituted with K21/22/24R mutant NLRP3 cleared glucose more effectively than the *Nlrp3*^-/-^ mice reconstituted with WT NLRP3. This indicated that SIRT2 and NLRP3 deacetylation prevent aging-associated inflammation and insulin resistance [131].

## 9. Regulation of S-Glutathionylation in NLRP3 Inflammasome Activation

Protein S-glutathionylation, an oxidative PTM, is a reversible formation of mixed disulfides between tripeptide glutathione and low-pKa cysteine [132]. Glutathione transferase Omega 1 (GSTO1-1) belongs to the cytosolic glutathione transferase (GST) super family [133]. *Gsto1-1* deletion reduces proinflammatory cytokine expression and ameliorates the inflammatory response in response to LPS in mice. GSTO1-1 deglutathionylates NEK7 Cys253 to promote its interaction with NLRP3 and the inflammasome activation [134]. Superoxide dismutase 1 (SOD1) contributes to caspase-1 activation via inhibiting its glutathionylation. Upon stimulation with LPS + ATP, SOD1 deficiency leads to increased ROS generation, decreased cellular redox potential and reversible oxidation and glutathionylation of caspase-1 Cys397 and Cys362, eliciting the inhibition of caspase-1 activity. Caspase-1 is activated and not glutathionylated in WT murine peritoneal macrophages [135]. Treatment with curcumin leads to a decrease in NLRP3 S-glutathionylation and an increase in caspase-1 S-glutathionylation, as well as the decreased production and secretion of IL-1β [136]. Administration of curcumin improves the survival of mice suffering from LPS-induced lethal endotoxic shock, and alleviates liver and kidney damage in mice [137].

## 10. The NLRP3 Inflammasome and Cancers

The NLRP3 inflammasome plays dual roles in the pathogenesis of cancers. It has a protective anti-tumorigenic effect in colitis-associated cancer, colorectal cancer, hepatocellular carcinoma and melanoma, and plays a pro-tumorigenic role in breast cancer, colon cancer, colorectal cancer, epithelial skin cancer, fibrosarcoma and gastric cancer [138,139]. Regulation of NLRP3 inflammasome activity via PTMs is essential for cancer development and progression. TRIM31 is upregulated at the protein level in human hepatocellular carcinoma and colorectal cancer, and promotes invasion and metastasis [140,141]. It mediates the K48-linked ubiquitination and degradation of NLRP3, restricting NLRP3 inflammasome activity [35]. Alpinumisoflavone and estrogen suppress the proliferation and metastasis of hepatocellular carcinoma cells via enhancing NLRP3 inflammasome activation [142,143]. Caffeic acid phenethyl ester (CAPE) enhances NLRP3 ubiquitination via facilitating NLRP3–Cullin1 interaction and suppresses NLRP3 inflammasome activation, which protects mice from azoxymethane/dextran sulfate sodium-induced colon cancer [39,144]. 5-hydroxytryptamine (5-HT) expression is upregulated in colorectal tumor tissues from patients with colorectal cancer, the azoxymethane/dextran sodium sulfate-induced colorectal cancer mouse model and colorectal cancer cell lines. 5-HT induces NLRP3 phosphorylation at Ser198 (mouse Ser194) and IL-1β release via its ion channel receptor HTR3A. 5-HT production is further promoted by elevated IL-1β levels in colorectal cancer cells, forming a 5-HT–NLRP3 positive feedback loop. HTR3A inhibition impairs tumor growth in vivo and in vitro [145]. The androgen receptor pathway is critical for the tumorigenesis of prostate cancer. CircAR-3, a circRNA derived from the androgen receptor gene, contributes to NLRP3 acetylation by KAT2B and NLRP3 inflammasome activation. Disturbing NLRP3 acetylation with the KAT2B inhibitor NS-1502 suppresses the progression of prostate cancer xenograft tumors [146].

## 11. Concluding Remarks

Aberrant NLRP3 inflammasome activation contributes to the pathogenesis of several inflammatory diseases. The fine orchestration of NLRP3 inflammasome activation is critical for maintaining proper cellular homeostasis and health. NLRP3 inflammasome activity is regulated at transcriptional, post-transcriptional and post-translational levels. MicroRNAs, including miR-223, miR-22, miR-30e, miR-7 and miR-133b, target the 3′-untranslated regions of NLRP3 to decrease their protein level, leading to the reduced inflammasome activity [147,148]. A variety of proteins are involved in abundant PTMs of the components of the NLRP3 inflammasome to alter the protein functions, activities and/or intracellular locations, and consequently regulating NLRP3 inflammasome activity. PTM crosstalk makes it more complicated. PKA-mediated NLRP3 phosphorylation may facilitate K48- and K63-linked ubiquitination, which causes the degradation. NLRP3 Ser194 phosphorylation by JNK1 promotes its deubiquitination. How some types of PTM affect NLRP3 inflammasome activity needs to be further explored, including alkylation, S-nitrosylation, acetylation and S-glutathionylation. Regulation of NLRP3 inflammasome activity by PTMs provides new targets for the prevention and therapy of NLRP3-associated diseases.

## Figures and Tables

**Table 1:** Ubiquitination and deubiquitination of NLRP3 inflammasome components.

Protein	Interacting Protein	Type	Regulation	Ubiquitin Chain Types	Sites	Interacting Domains	References
MARCH7	NLRP3	Ubiquitination	Inhibition	K48	?	LRR	[33]
Cbl-b	NLRP3	Ubiquitination	Inhibition	K48	Lys496	NACHT	[34]
TRIM31	NLRP3	Ubiquitination	Inhibition	K48	?	PYD	[35]
ARIH2	NLRP3	Ubiquitination	Inhibition	K48/K63	?	NACHT	[36]
TRIM65	NLRP3	Ubiquitination	Inhibition	K48/K63	?	NACHT	[37]
Pellino-2	NLRP3	Ubiquitination	Promotion	K63	?	?	[38]
Cullin1	NLRP3	Ubiquitination	Inhibition	K63	Lys689	?	[39]
Ubc13	NLRP3	Ubiquitination	Promotion	K63	Lys565	?	[40]
β-TrCP1	NLRP3	Ubiquitination	Inhibition	K27	Lys380	NACHT	[41]
HUWE1	NLRP3	Ubiquitination	Promotion	K27	Lys21/Lys22/Lys24	NACHT	[42]
SCF-FBXL2	NLRP3	Ubiquitination	Inhibition	?	Lys689	PYD Trp73	[43]
STING	NLRP3	Deubiquitination	Promotion	K48/K63	?	?	[44]
UAF1	NLRP3	Deubiquitination	Promotion	K48	?	LRR, NACHT	[45]
UCHL5	NLRP3	Deubiquitination	Promotion	K63	?	?	[46]
STAMBP	NLRP3	Deubiquitination	Inhibition	K63	?	?	[47]
TRAF3	ASC	Ubiquitination	Promotion	K63	Lys174	?	[48]
Peli1	ASC	Ubiquitination	Promotion	K48, K63	Lys55	?	[49]
USP8	caspase-1	Deubiquitination	Promotion	K11	Lys134	p20	[50]

**Table 2:** Phosphorylation and dephosphorylation of NLRP3 inflammasome components.

**Protein**	**Interacting Protein**	**Regulation**	**Type**	**Site**	**References**
MINK1	NLRP3	Promotion	Phosphorylation	Ser725	[81]
JNK1	NLRP3	Promotion	Phosphorylation	Ser198	[82]
AKT	NLRP3	Inhibition	Phosphorylation	Ser5	[83]
PP2A	NLRP3	Promotion	Dephosphorylation	Ser5	[84]
PTPN22	NLRP3	Promotion	Dephosphorylation	Tyr861	[85]
PKD	NLRP3	Promotion	Phosphorylation	Ser295	[66]
PKA	NLRP3	Inhibition	Phosphorylation	Ser295	[86]
Pyk2	ASC	Promotion	Phosphorylation	Tyr146	[87]
PAK1	caspase-1	Promotion	Phosphorylation	Ser376	[88]

## Data Availability

Not applicable.
